# Editorial: Impression Management and Faking in Job Interviews

**DOI:** 10.3389/fpsyg.2017.01294

**Published:** 2017-07-28

**Authors:** Joshua S. Bourdage, Nicolas Roulin, Julia Levashina

**Affiliations:** ^1^Department of Psychology, University of Calgary Calgary, AB, Canada; ^2^Asper School of Business, University of Manitoba Winnipeg, MB, Canada; ^3^College of Business Administration, Kent State University Kent, OH, United States

**Keywords:** employment interviews, personnel selection, impression management, interview faking behavior, self-presentation, industrial psychology, organizational psychology, job interviews

Organizations place a great deal of emphasis on hiring individuals who are a good fit for the organization and the job. Among the many ways that individuals are screened for a job, the employment interview is particularly prevalent and nearly universally used (Macan, 2009; Huffcutt and Culbertson, 2011). This Research Topic is devoted to a construct that plays a critical role in our understanding of job interviews: impression management (IM). In the interview context, IM describes behaviors an individual uses to influence the impression that others have of them (Bozeman and Kacmar, 1997). For instance, a job applicant can flatter an interviewer to be seen as likable (i.e., ingratiation), play up their qualifications and abilities to be seen as competent (i.e., self-promotion), or utilize excuses or justifications to make up for a negative event or error (i.e., defensive IM; Ellis et al., 2002). IM has emerged as a central theme in the interview literature over the last several decades (for reviews, see Posthuma et al., 2002; Levashina et al., 2014). Despite some pioneering early work (e.g., Schlenker, 1980; Leary and Kowalski, 1990; Stevens and Kristof, 1995), there has been a resurgence of interest in the area over the last decade. While the literature to date has set up a solid foundational knowledge about interview IM, there are a number of emerging trends and directions. In the following, we lay out some critical areas of inquiry in interview IM, and highlight how the innovative set of papers in this Research Topic is illustrative of these new directions.

## Honest and deceptive IM

In understanding the content domain of IM, a critical distinction to make is whether IM is honest or deceptive (i.e., faking). For example, as Levashina and Campion (2006, 2007) illustrate, applicants can promote their skills and abilities both honestly and deceptively. Similarly, in using ingratiation, applicants could flatter the organization on admirable qualities, but this flattery may be based in truth (i.e., they actually find this organization to be admirable) or deceptive (i.e., they don't really care about these qualities). Interviewers generally find honest IM to be more acceptable and deceptive IM less acceptable (Jansen et al., 2012). Importantly, from a measurement perspective, observers tend to be quite poor at identifying and distinguishing honest and deceptive IM (Roulin et al., 2015), and a recent review of the interview literature notes that some self-report measures confound the two (Levashina et al., 2014). Roulin and Bourdage's study demonstrates that honest and deceptive IM have different antecedents and are engaged in by applicants with different sorts of traits. In other words, in order to truly understand the domain of IM behavior, we must consider both sides of IM. Yet, more research is needed, for instance to examine how honest vs. deceptive IM impacts interview validity.

## Antecedents of IM

Perhaps the largest body of literature to date has examined antecedents of IM. Consistent with the political influence perspective (Ferris and Judge, 1991), antecedents can broadly be understood in terms of (a) actor characteristics, (b) situational characteristics, and (c) target characteristics. In the interview IM literature, there has been a particular focus on actors' characteristics such as personality (Kristof-Brown et al., 2002; Higgins and Judge, 2004). This trend is reflected in three studies in this Research Topic, which examine a variety of individual differences, including personality traits (e.g., Honesty-Humility and Extraversion), intelligence, and social skill (Law et al.; Buehl and Melchers; Roulin and Bourdage). However, additional research is needed to examine the situational and target antecedents of IM use. The Law et al. study adds to our understanding of situational antecedents by demonstrating that identification warnings against faking can reduce deceptive IM use. From a practical perspective, this demonstrates that deceptive IM can be reduced through manipulation of the context. Although encouraging, more work is needed on various contextual factors and interview practices that may deter deceptive IM. Moreover, work on target characteristics and IM have been very sparse. Extant work (e.g., Delery and Kacmar, 1998) has found interviewer characteristics such as communication skill or experience can influence which IM tactics are used, but future work should expand the contextual and target antecedents of IM.

## Use of IM vs. effectiveness of IM

Three papers in this Research Topic (Law et al.; Buehl and Melchers; Roulin and Bourdage) demonstrate that individuals who are low in trait Honesty-Humility are more likely to engage in deceptive IM. This is particularly problematic if deceptive IM leads to better interview performance. However, although the meta-analysis by Peck and Levashina demonstrates a positive effect of IM on interview ratings, primary studies honing in specifically on deceptive IM have been more mixed. One possibility is that there are factors that impact the effectiveness of IM. As such, it is important to distinguish between IM use and IM effectiveness.

In line with this important distinction, Buehl and Melchers investigate the idea that some individuals may be more effective at using deceptive IM than others. Across two studies, they examined the roles of intelligence and social skills on deceptive IM effectiveness. Although they did not find support for these relationships, future research should continue to examine individual differences that might impact IM effectiveness. Research on workplace IM has found that individuals high in political skill, for instance, are more effective IM users (e.g., Harris et al., 2007). Peck and Levashina found that current labor market participants used IM slightly more frequently and significantly more effectively than students. Finally, moving beyond individual differences of the actor, it is likely important to determine situational and target characteristics that impact IM effectiveness. As Derous demonstrated, different raters likely have difference preferences surrounding IM, and recent research by Roulin (2016) has demonstrated that some interviewers are better at detecting deceptive IM than others.

Finally, there is room for further investigation of the cognitive and affective processes by which IM impacts interviewer ratings. From a cognitive perspective, Bozeman and Kacmar (1997) illustrate a number of potentially important processes. Moreover, work suggests that IM behaviors vary in how acceptable they are viewed by interviewers (Jansen et al., 2012), which may have important implications for understanding why some IM behaviors are rewarded and others are not. From an affective perspective, IM tactics are proposed to work at least somewhat by creating an impression that one is similar to the interviewer (Ferris and Judge, 1991), and attributions of likability (Wayne and Liden, 1995). More research could thus investigate the mechanisms by which applicant IM helps trigger positive emotions or building a positive relationship with the interviewer, ultimately leading to higher ratings.

## IM as dyadic and beyond the applicant

Although the IM literature has almost exclusively focused on how applicants use IM toward interviewers to get hired, this misses a fundamental aspect of the nature of IM. IM takes place in the context of at least a two-way interaction. Peck and Levashina showed that interview ratings were saturated more with IM when the raters were the targets of IM rather than observers. Applicants are indeed trying to influence interviewers, but interviewers are also reacting to and attempting to influence applicants, and thus also use IM. The study by Wilhelmy et al. is rare in examining the role of interviewer IM, joining only two other published studies in this area (Stevens et al., 1990; Wilhelmy et al., 2016). Drawing on signaling theory, these authors present a model demonstrating that interviewer use of IM can ultimately impact perceptions of the organization (i.e., organizational prestige) and applicants' positive affect and feelings (i.e., interview self-efficacy). Future research should continue to consider IM from multiple perspectives.

## IM as a shield against discrimination

One additional theme that is highlighted in the current Research Topic is interplay between IM and diversity or discrimination. Derous' study considers the cultural context surrounding IM. She notes that with increasingly multicultural workplaces, understanding the role of culture in IM is particularly important, although greatly under-researched (e.g., Bolino et al., 2016). She demonstrates that culture can impact preferences for different IM tactics, and that minority applicants might be discriminated against, unless they use majority-preferred IM tactics. This article also highlights the importance for future work considering IM in a cross-cultural context. Gioaba and Krings' study highlights the role of age. These authors use the idea of “social-identity based IM” (Roberts, 2005), which proposes that one can use IM to refute negative stereotypes associated with one's social group. Gioaba and Krings' work suggests that older individuals can use IM to somewhat mitigate the effects of discrimination—although all things being equal, younger applicants were still favored. Nonetheless, both articles highlight that IM can have a positive impact for candidates potentially facing discrimination.

## IM across time

Finally, one area that should receive increased attention is the temporal nature of IM. For instance, although job applicants typically participate in multiple interviews, IM research typically asks individuals to rate their use of IM in a single interview, or in general across interviews (Buehl and Melchers). Consistent with models suggesting that applicant faking behavior is likely dynamic, Roulin and Bourdage measured applicant IM use across several interviews, finding that IM use varies substantially across interviews, and that different individual differences explain this variability. For instance, applicants high in Psychopathy adapted their IM use more. Future research should continue to explore IM across multiple interviews, and the dynamic of IM use over time. Research can also continue to delve into how IM use and its impact on interviewers' ratings unfolds within an interview. For instance, IM effectiveness partly depends on the first impression applicants make on interviewers (Swider et al., 2011), but more research could examine if IM is more effective when used early vs. later on in an interview.

## Conclusion

In conclusion, it is an exciting and interesting time to be doing research into interview IM. The set of studies presented in this Research Topic demonstrates a number of new and emerging trends that should bolster the area for years to come.

## Author contributions

Each author contributed to the writing and conceptualization of this Editorial. The order of contribution is in line with the authorship order.

### Conflict of interest statement

The authors declare that the research was conducted in the absence of any commercial or financial relationships that could be construed as a potential conflict of interest.
